# EHPnet: Green Halloween

**Published:** 2008-10

**Authors:** Erin E. Dooley

http://www.greenhalloween.org/

Halloween—what would it be without all the candy children pick up door to door? But much of the candy that’s typically distributed at Halloween contains high amounts of *trans* fats, high-fructose corn syrup, sodium, and dyes. Green Halloween, launched in 2007 by a Seattle mother, is a grass-roots movement that encourages parents, children, schools, and communities to make Halloween a healthier and more sustainable holiday.

The Parents section of the Green Halloween website provides ideas on how to select affordable healthier treats to give out at Halloween—including non-candy alternatives—with an emphasis on selecting organic and locally produced items with minimal packaging. The Kids section includes ideas for alternatives to trick-or-treating for candy, among them the traditional UNICEF coin collection program as well as Reverse Trick-or-Treating, an initiative of Global Exchange that seeks to educate the public about sustainable and ethical cocoa production. The Help Out page lists other ways to celebrate Green Halloween, for example by hosting a neighborhood party and choosing decorations that use natural products and help eliminate waste.

